# Perfusion Capacity as a Predictive Index for Assessing Visual Functional Recovery in Patients With Idiopathic Epiretinal Membrane

**DOI:** 10.1167/tvst.14.1.19

**Published:** 2025-01-21

**Authors:** Jinlian Zhan, Chen Chen, Tong Wang, Qi Zhang, Xia Huang, Lin Lu, Xiujuan Zhao

**Affiliations:** 1 State Key Laboratory of Ophthalmology, Zhongshan Ophthalmic Center, Sun Yat-Sen University, Guangdong Provincial Key Laboratory of Ophthalmology and Visual Science, Guangdong Provincial Clinical Research Center for Ocular Diseases, Guangzhou, China

**Keywords:** idiopathic epiretinal membrane (iERM), optical coherence tomography angiography (OCTA), superficial vascular complex (SVC), perfusion capacity (PC), retinal sensitivity

## Abstract

**Purpose:**

This study investigates the association between visual function and retinal vasculature metrics, particularly perfusion capacity (PC), in eyes with idiopathic epiretinal membrane (iERM), using optical coherence tomography angiography (OCTA).

**Methods:**

This retrospective study includes 30 eyes from 30 iERM patients who had surgery, with a three-month follow-up period. In addition, 28 eyes from 28 healthy individuals served as a control group. We measured best-corrected visual acuity (BCVA), retinal sensitivity, vascular density (VD), perfusion area (PA), PC, and retinal thickness (RT). OCTA scans assessed both the superficial vascular complex (SVC) and deep vascular complex (DVC) in 3 × 3 mm^2^ and 6 × 6 mm^2^ regions. Associations between retinal vasculature metrics and visual outcomes were analyzed.

**Results:**

Postoperatively, significant improvements were observed in both BCVA and retinal sensitivity (both *P* < 0.001). In the SVC layer, VD and PA were significantly decreased (both *P* < 0.001), whereas PC in the SVC increased significantly (*P* < 0.001). Higher preoperative PC in the SVC was associated with greater postoperative improvements in retinal sensitivity. Postoperative retinal sensitivity was negatively associated with age (β = −0.53, *P* = 0.001) and retinal thickness in 3 × 3 mm^2^ region (*β* = −0.39, *P* = 0.013) but positively associated with preoperative retinal sensitivity (*β* = 1.10, *P* < 0.001) and PC in the SVC within 3 × 3 mm^2^ region (*β* = 0.49, *P* = 0.023).

**Conclusions:**

PC offers a novel approach to evaluating retinal microcirculation and visual prognosis in iERM. Preoperative PC in the SVC serves as a reliable predictive index for predicting postoperative visual recovery.

**Translational Relevance:**

The PC, as a novel indicator of retinal blood flow, not only reflects the condition of the blood vessels but is also associated with retinal sensitivity.

## Introduction

Idiopathic epiretinal membrane (iERM) is a condition characterized by fibrocellular tissue formation at the vitreoretinal interface, which induces retinal architecture deformation, including thickening and foldingd.^1^^,^^2^ Age is a contributing factor to the 2.2% to 28.9% incidence of iERM.^3^ The structural alteration, especially in the macula, leads to visual dysfunction, with metamorphopsia and blurred central vision being common symptoms.^2^^,^^4^ Despite its significant impact on visual quality, iERM often presents with a relatively good best-corrected visual acuity (BCVA) in the early stages, masking underlying retinal dysfunction. This makes BCVA an imperfect measure for visual recovery after surgery, particularly in cases with subtle macular changes.

Pars plana vitrectomy, an effective method for the treatment of iERM, aims to remove the epiretinal membrane and restore retinal architecture. Although BCVA recovery after surgery is frequently reported, it does not fully capture the extent of visual impairment, particularly when subtle functional deficits remain. Notably, many patients with iERM retain relatively good BCVA despite experiencing blurred vision, metamorphopsia, and difficulty distinguishing objects in low light or low-contrast environments. Microperimetry, which measures retinal sensitivity, although less frequently used in iERM, offers a more sensitive method for detecting early functional changes^5^ and has shown improved reliability over traditional visual acuity tests,^6^ making it an ideal tool for evaluating macular function in these patients.

With the advent of optical coherence tomography angiography (OCTA), a more detailed, depth-resolved imaging modality, there has been growing interest in quantifying retinal blood flow and microvascular changes in diseases like iERM.^7^^,^^8^ OCTA allows differentiation between the superficial vascular complex (SVC) and deep vascular complex (DVC),^9^^,^^10^ and several indexes such as foveal avascular zone parameters,^11^ vascular density (VD), fractal dimension,^12^ and vessel tortuosity^13^ have been explored. However, these measures are limited in their ability to fully capture the dynamic changes in retinal perfusion, particularly in conditions where there is significant structural deformation or vascular remodeling, as seen in iERM.

To address this gap, we introduce a new metric, vascular perfusion capacity (PC), which combines both vascular density and perfused area, providing a more comprehensive and dynamic assessment of retinal perfusion. Unlike VD, which may be influenced by macular edema or vascular congestion, PC reflects the actual blood flow capacity, accounting for both vessel dilation and microvascular impairment.

Therefore this study aimed to evaluate both preoperative and postoperative visual acuity in patients with iERM, alongside the assessment of retinal vascular parameters using OCTA, which has not been extensively applied in iERM.^13^^–^^16^ Furthermore, we analyzed the correlation between preoperative retinal vascular metrics and postoperative visual outcomes to elucidate the impact of microvascular alterations associated with iERM on visual function.

## Methods

### Participants

This retrospective study involved 30 eyes of 30 patients with iERM and 28 eyes of 28 healthy volunteers at the Zhongshan Ophthalmic Center from March 2023 to February 2024.The study was approved by the Zhongshan Ophthalmic Center Ethics Committee and adhered to the principles of the Declaration of Helsinki. A senior surgeon (LL) performed all procedures, including 25-gauge pars plana vitrectomy, iERM peeling, and non-foveal-sparing internal limiting membrane peeling. All surgeries were completed without intraoperative complications. Phacoemulsification and intraocular lens implantation were performed on patients over 55 years of age with mild cataracts.

The inclusion criteria were as follows: (1) iERM diagnosed by retinal specialists using fundus examination, and OCT; (2)the axial length of all included eyes ranged from 22.00 mm to 25.00 mm; and (3) a minimum follow-up period of three months. Exclusion criteria included secondary ERM associated with other retinal diseases, severe cataract or glaucoma, high myopia (refractive error ≤ −6.00 diopters or axial length > 26.0 mm), retinal vascular diseases, prior vitreoretinal surgery, uveitis, or uncontrolled systemic disease. All participants underwent comprehensive pre- and postoperative evaluations, including BCVA, retinal sensitivity, and OCTA imaging.

### Sample Size Estimation

To confirm the adequacy of our sample size, we conducted a paired-sample power analysis on pre- and postoperative retinal sensitivity and the PC in the SVC within 3 × 3 mm^2^ region. Using PASS 2021 software (NCSS, LLC, Kaysville, UT, USA),^17^^,^^18^ designed for power and sample size calculations, we set a significance level of (α = 0.05)and a power of 0.9.For retinal sensitivity, a sample size of 11 pairs achieves 91.6% power to reject the null hypothesis using a two-sided paired *t*-test. For PC, a sample size of 12 pairs achieves 91.6% power. Consequently, we ultimately enrolled approximately 30 patients in the study to ensure an adequate sample size.

### Functional Assessment

BCVA was measured using a Snellen chart, with values converted to the logMAR for statistical analysis. Retinal sensitivity was assessed with the Nidek MP-3 microperimeter (Nidek Co., Tokyo, Japan), using a 200-ms Goldmann III (25.7-minarc) stimulus. The test used a 4-2-1 automatic staircase strategy, ranging from 0 to 20 dB in 2 dB increments. The fixation target was a 1-mm red ring on a white monochrome background set at 31.4 abs. Retinal sensitivity was calculated as the mean sensitivity across 33 points (covering a 3 × 3-mm^2^ area) within the central 10° of the fovea, with a dynamic range of 34 dB.

### Central Retinal Thickness (RT) Assessment and iERM Stage

Mean retinal thickness in the macular area (within a 3-mm diameter) and the paramacular region (within a 6-mm diameter) was measured using the swept-source OCT/OCTA system. To evaluate preoperative and postoperative changes, full central retinal thickness was assessed. The iERM stage was classified according to the staging system proposed by Govetto et al.^3^

### OCTA Imaging

OCTA scans were acquired using a swept-source OCT/OCTA system (VG200; S Vision Imaging, Luoyang, China). This instrument used a central wavelength of 1050 nm (990–1100 nm full width) and an A- scan rate of 200,000/s. The OCTA vascular metrics in this study were derived from the en face OCTA images, rather than OCTA B-scans. OCTA provides direct image, VD and perfusion area (PA) of the retina. VD and PA of the retina, including SVC and DVC, were automatically calculated in the inner 3 mm and 6 mm circles of the Early Treatment of Diabetic Retinopathy Study chart. The SVC layer extended from 5 µm above the internal limiting membrane to the upper third of the retinal ganglion cell complex, whereas the DVC layer spanned from the upper third of the retinal ganglion cell complex to 25 µm below the outer plexiform layer.^19^ After automated segmentation, a retinal specialist verified the accuracy of segmentation for all images. VD was calculated as the percentage of blood vessel area in the scanned region, whereas PA represented the perfused area within the region.

### Image Analysis

PC was defined as the ratio of the perfusion area to the total area, adjusted for vascular density, providing a continuous measure of perfusion efficiency (Fig. A). An abstract illustration of blood vessels is provided in Figure B.

**Figure.**
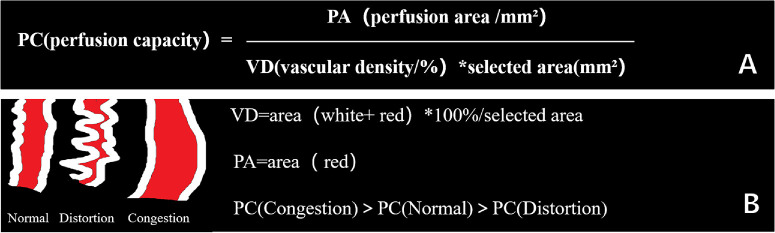
Processing step for analyzing PC. (**A**) Perfusion capacity equals to the ratio of the PA in a selected region to the area of that region, which is multiplied by its VD. (**B**) The abstract metaphor of blood vessels. PA, perfusion area; PC, perfusion capacity; VD, vascular density.

### Statistical Analysis

All statistical analyses were conducted using the Statistical Package for the Social Sciences (SPSS) version 25.0 (SPSS Inc., Chicago, IL, USA). Continuous variables are presented as mean ± standard deviation (SD). To those with non-normal distribution, we applied the Mann-Whitney U test and Wilcoxon signed-rank test. All data followed a normal distribution. Group differences were assessed using independent-sample *t*-tests, and paired *t*-tests were used to compare preoperative and postoperative retinal sensitivity, BCVA (logMAR), and anatomical parameters.

Pearson correlation analysis (or Spearman's rank correlation coefficient, as appropriate) was performed to examine the relationships between pre-operative anatomical parameters and changes in visual parameters. Multiple linear regression was used to analyze the influence of various factors on post-operative visual function. Statistical significance was set at *P* < 0.05.

## Results

### Baseline Information

The baseline demographic and clinical characteristics of the iERM and control groups are summarized in Table 1. The iERM group included 30 eyes (13 males, 17 females; mean age, 62.30 ± 9.11 years; axial length, 23.67 ± 0.90mm) with iERM stages distributed as follows: stage 2, 9 eyes; stage 3, 13 eyes; and stage 4, eight eyes. The control group included 28 healthy eyes (12 males, 16 females; mean age, 61.14 ± 4.63 years; axial length, 23.72 ± 0.73 mm). No significant differences were found between the two groups in terms of age (*P* = 0.549), gender (*P* = 1.000) or axial length (*P* = 0.162).

**Table 1.** Demographics and Ocular Characteristics of the Study Population

	Control Group (N = 28)		iERM Group (N = 30)			
Variables	3 × 3 mm^2^	6 × 6 mm^2^	3 × 3 mm^2^	6 × 6 mm^2^	*P*1^*^	*P*2^†^
Age (years)	61.14 ± 4.63		62.30 ± 9.11		0.549	
Gender					1.000	
Male	12		13			
Female	16		17			
Axial length (mm)	23.72 ± 0.73		23.67 ± 0.90		0.162	
BCVA (logMAR)	0.12 ± 0.10		0.55 ± 0.23		**<0.001**	
Retinal Sensitivity (dB)	26.60 ± 0.78		21.83 ± 3.88		**<0.001**	
ERM Stage						
Stage 2	—		9 (30.00%)		—	
Stage 3	—		13 (43.33%)		—	
Stage 4	—		8 (26.67%)		—	
Anatomic parameters						
RT (µm)	312.58 ± 25.45	289.02 ± 14.78	460.99 ± 60.73	368.08 ± 36.70	**<0.001**	**<0.001**
VD (%)						
SVC	31.79 ± 8.23	39.09 ± 6.28	49.86 ± 14.89	51.08 ± 8.99	**<0.001**	**<0.001**
DVC	23.53 ± 5.91	21.38 ± 6.18	21.29 ± 6.11	20.06 ± 4.58	0.162	0.357
PA (mm^2^)						
SVC	2.24 ± 0.51	10.80 ± 1.56	3.35 ± 0.95	13.91 ± 2.33	**<0.001**	**<0.001**
DVC	1.77 ± 0.43	6.72 ± 1.73	1.63 ± 0.38	6.10 ± 1.25	0.177	0.121
PC						
SVC	0.79 ± 0.03	0.77 ± 0.03	0.75 ± 0.03	0.76 ± 0.02	**<0.001**	0.069
DVC	0.85 ± 0.08	0.90 ± 0.15	0.88 ± 0.15	0.85 ± 0.07	0.282	0.115

* Comparison between iERM eyes and control eyes within 3 × 3 mm^2^.

† Comparison between iERM eyes and control eyes within 6 × 6 mm^2^.

### Preoperative Comparisons

At baseline, iERM eyes exhibited significantly lower retinal sensitivity and BCVA compared to healthy controls, as expected (*P* < 0.001 for both measures, Table 1). Preoperative retinal thickness was significantly greater in both the 3 × 3 mm^2^ and 6 × 6 mm^2^ regions in the iERM group (both *P* < 0.001), consistent with the macular swelling associated with the condition. Interestingly, while VD and PA in the SVC were significantly elevated in the iERM group (all *P* < 0.001), PC in the SVC was significantly lower in the 3 × 3 mm^2^ region (*P* < 0.001).No significant differences were detected in the DVC layer (*P* > 0.05).

### Postoperative Changes

Postoperative analysis revealed significant improvements in both BCVA and retinal sensitivity (*P* < 0.001 for both, Table 2). The average improvement in BCVA was −0.19 ± 0.21 logMAR, whereas retinal sensitivity increased by 2.85 ± 2.54 dB. Retinal thickness in both the 3 × 3 mm^2^ and 6 × 6 mm^2^ regions showed significant reductions postoperatively (*P* < 0.001 for both regions). After the removal of the iERM, the restoration of retinal perfusion was observed in the SVC layer, where postoperative VD and PA were significantly lower compared to baseline (both *P* < 0.001), whereas PC in the SVC significantly increased in both the 3 × 3 mm^2^ and 6 × 6 mm^2^ regions after surgery (both *P* < 0.001). No significant changes were observed in the DVC layer (*P* > 0.05).

**Table 2.** Functional and Anatomic Parameters at Baseline and Three Months After Surgery in the iERM Group

	Baseline		Three Months				
iERM Group (N = 30)	3 × 3 mm^2^	6 × 6 mm^2^	3 × 3 mm^2^	6 × 6 mm^2^	*P*1^*^	*P*2^†^	Change
BCVA (logMAR)	0.55 ± 0.23		0.36 ± 0.17		**<0.001**		−0.19 ± 0.21
Retinal Sensitivity (dB)	21.83 ± 3.88		24.68 ± 3.03		**<0.001**		2.85 ± 2.54
Anatomic parameters							
RT (µm)	460.99 ± 60.73	368.08 ± 36.70	392.32 ± 34.16	340.01 ± 21.74	**<0.001**	**<0.001**	
VD (%)							
SVC	49.86 ± 14.89	51.08 ± 8.99	35.82 ± 11.82	40.87 ± 7.31	**<0.001**	**<0.001**	
DVC	21.29 ± 6.11	20.06 ± 4.58	23.27 ± 8.91	22.28 ± 6.64	0.332	0.062	
PA (mm^2^)							
SVC	3.35 ± 0.95	13.91 ± 2.33	2.51 ± 0.75	11.39 ± 1.92	**<0.001**	**<0.001**	
DVC	1.63 ± 0.38	6.10 ± 1.25	1.71 ± 0.61	6.60 ± 1.81	0.528	0.130	
PC							
SVC	0.75 ± 0.03	0.76 ± 0.02	0.79 ± 0.04	0.78 ± 0.03	**<0.001**	**<0.001**	
DVC	0.88 ± 0.15	0.85 ± 0.07	0.85 ± 0.12	0.84 ± 0.07	0.353	0.316	

^*^Comparison between post-operative and pre-operative iERM eyes within 3 × 3-mm^2^.

^†^Comparison between post-operative and pre-operative iERM eyes within 6 × 6-mm^2^.

### Correlations Between Preoperative Anatomical Parameters and Visual Function Improvement

Baseline factors associated with changes in retinal sensitivity included 3 × 3 mm^2^ RT (*r* = −0.389, *P* = 0.034,Table 3) and PC in both the 3 × 3 mm^2^ and 6 × 6 mm^2^ SVC regions (3 × 3 mm^2^ SVC PC: *r* = 0.71, *P* < 0.001; 6 × 6 mm^2^ SVC PC: *r* = 0.53, *P* = 0.003; Table 3). No significant correlations were found between baseline VD or PA and postoperative improvements in retinal sensitivity.

**Table 3.** Correlations Between Preoperative Anatomical Parameters and Visual Function Improvement at Three Months After iERM Surgery

	Change in BCVA (logMAR)				Change in Retinal Sensitivity (dB)			
	3 × 3 mm^2^		6 × 6 mm^2^		3 × 3 mm^2^		6 × 6 mm^2^	
Variables	*R*	*P*	*R*	*P*	*R*	*P*	*R*	*P*
RT (µm)	−0.18	0.331	−0.12	0.534	−0.39	**0.034**	−0.35	0.061
SVC								
VD (%)	−0.31	0.099	−0.21	0.269	−0.32	0.084	−0.31	0.100
PA (mm^2^)	−0.34	0.070	−0.25	0.184	−0.29	0.123	−0.27	0.145
PC	0.01	0.960	−0.21	0.273	0.71	**<0.001**	0.53	**0.003**

### Multiple Linear Regression Analysis

In multiple linear regression analysis, preoperative PC in the SVC within the 3 × 3 mm^2^ region was the strongest predictor of postoperative retinal sensitivity (*r* = 0.71, *P* < 0.001). The regression model showed that postoperative retinal sensitivity was negatively associated with age (*β* = −0.53, *P* = 0.001) and 3 × 3 mm^2^ RT (*β* = −0.39, *P* = 0.013), while positively associated with preoperative retinal sensitivity (*β* = 1.10, *P* < 0.001) and PC in the SVC within 3 × 3 mm^2^ region (*β* = 0.49, *P* = 0.023; Table 4).

**Table 4.** Multiple Linear Regression Model of Postoperative Retinal Sensitivity and Baseline Ocular Parameters (N = 30)

	Postoperative Retinal Sensitivity (dB)			
Baseline Parameters	β	SE (β)	*P*	95% Cl
Age (years)	−0.18	−0.53	**0.001**	−0.27 to −0.08
Gender	−0.15	−0.03	0.811	−1.41 to 1.12
Pre-operative retinal sensitivity (dB)	0.86	1.10	**<0.001**	0.59 to 1.13
3 × 3 mm^2^ RT (µm)	−0.02	−0.39	**0.013**	−0.03 to −0.00
3 × 3 mm^2^ SVC PC	55.01	0.49	**0.023**	8.24 to 101.78
3 × 3 mm^2^ SVC VD (%)	0.01	0.04	0.777	−0.05 to 0.07

SE (β), standard error of β coefficient.

Adjusted *R^2^* for postoperative retinal sensitivity = 0.748.

### Correlations Between Postoperative Anatomical Parameters and Postoperative Visual Outcomes

Postoperative BCVA (logMAR) was significantly correlated with postoperative PC in the 6 × 6 mm^2^ SVC region (*r* = −0.42, *P* = 0.021; Supplementary Table S1).

## Discussion

This study demonstrates that PC serves as a valuable and innovative evaluation index for assessing retinal microcirculation in patients with iERM, as well as for predicting postoperative recovery of visual acuity and retinal sensitivity. In contrast to conventional assessment metrics such as VD and PA, PC provides a more nuanced and dynamic evaluation of iERM.

Those with higher preoperative PC of the SVC tended to experience greater improvements in retinal sensitivity and achieved higher postoperative sensitivity levels. So, we suspect that when the iERM causes more severe vascular distortion and deformation in the SVC layer, the PC value of the SVC decreases, and the increased vascular resistance in the SVC layer leads to greater congestion and dilation in the DVC layer, which raises the PC value of the DVC. The more the vascular condition deviates from normal, the greater the damage to the retina. Only when the retinal vascular PC is within the normal range does the retinal vasculature maintain a stable state. We suspect that enhancing retinal perfusion preoperatively might improve surgical outcomes. This opens avenues for adjunct therapies that could be administered prior to surgery to optimize the microvascular environment.

Traditional OCTA metrics like VD and PA have limitations in accurately reflecting the functional status of retinal vasculature, especially in conditions like iERM where structural distortion is significant. VD can be confounded by factors such as macular edema,^20^ vessel compression, and tortuosity, which do not necessarily correlate with perfusion efficiency.^16^^,^^21^ Previous research have presented varying results about the relationships between retinal vascular parameters and visual function in iERM. Bacherini et al.^15^ discovered that lower VD in both the SVC and the DVC connected with poorer visual acuity, whereas Yuce et al.^16^ showed that higher VD in the DVC correlated with poorer visual acuity. However, other study^22^ and our study showed that neither VD nor PA correlated significantly with visual function outcomes, aligning with prior research that highlighted the limitations of these parameters. Moreover, the inconsistency in previous findings regarding changes in VD of the SVC and DVC in iERM eyes, with some studies indicating an increase, decrease, or no significant change, adding to the confusion.^12^^,^^13^^,^^22^^,^^23^ Our findings suggest that PC, by integrating both VD and PA into a single metric that reflects the actual perfusion capacity of retinal vessels, provides a more accurate representation of microvascular function.

Recent studies have highlighted the importance of considering vessel caliber and flow dynamics rather than just vessel density. For instance, research by Tang et al.,^24^ emphasized the role of vessel diameter index reflecting the average vessel caliber over mere structural presence in diabetic retinopathy. Similarly, Nishigori et al. ^25^ identified a new OCTA metric, the variable interscan time analysis, which can detect macular perfusion changes may be associated with predicting the recurrence of macular edema in retinal vein occlusion. These studies suggest that it is essential to investigate the perfusion of blood vessels, supporting our approach of using PC as a more comprehensive metric.

Our study further highlights the differential impact of iERM on the SVC and DVC. The SVC, being closer to the vitreoretinal interface, is more directly affected by the mechanical traction of the iERM. This leads to significant alterations in PC within the SVC, which correlates with changes in retinal sensitivity. In contrast, the DVC appears to be less affected structurally but may experience functional changes due to altered perfusion dynamics. Recognizing these layer-specific effects not only enhances our understanding of iERM pathophysiology but also emphasizes the significance for targeted OCTA metrics, like PC, that can differentiate between the impacts on superficial and deep retinal vasculature.

The ability to predict postoperative visual outcomes using preoperative PC has significant clinical implications. Surgeons can use PC measurements to identify patients who are more likely to benefit from surgical intervention, thereby optimizing patient selection and managing expectations. Additionally, monitoring PC could help in assessing the efficacy of novel therapeutic approaches, such as pharmacological agents aimed at improving microvascular perfusion.

### Limitations

Although our study provides valuable insights, it is not without limitations. The follow-up period is limited to three months. Future studies with larger cohorts and longer follow-up periods are necessary to confirm the predictive value for long-term prognosis of PC. Moreover, incorporating other functional assessments, such as contrast sensitivity, metamorphopsia, and patient-reported outcome measures, could provide a more comprehensive understanding of visual function recovery. And future studies should further investigate the relationship between iERM stage and perfusion capacity, and how this may affect visual recovery.

## Conclusions

In conclusion, PC emerges as a novel and robust metric for evaluating retinal microcirculation in idiopathic epiretinal membrane. Our study demonstrates that preoperative PC in the superficial vascular complex is a significant predictor of postoperative retinal sensitivity improvement. This finding has important clinical implications, suggesting that PC can be used to better select candidates for surgery and to tailor personalized treatment plans.

## Supplementary Material

Supplement 1
