# Supplementary material for: Perfusion Capacity as a Predictive Index for Assessing Visual Functional Recovery in Patients With Idiopathic Epiretinal Membrane
Source: Transl Vis Sci Technol. 2025 Jan 21;14(1):19. doi: 10.1167/tvst.14.1.19 (PMC11756611; doi:10.1167/tvst.14.1.19)
Supplement: Supplement 1 [file tvst-14-1-19_s001.docx]

Supplementary Table1. Correlations between postoperative anatomical parameters and postoperative visual outcomes

| Variables | BCVA (logMAR) | | | | |  | Retinal Sensitivity (dB) | | | | |
| --- | --- | --- | --- | --- | --- | --- | --- | --- | --- | --- | --- |
|  | 3 × 3-mm² | |  | 6 × 6-mm² | |  | 3 × 3-mm² | |  | 6 × 6-mm² | |
|  | R | *P* |  | R | *P* |  | R | *P* |  | R | *P* |
| RT(μm） | 0.07 | 0.726 |  | 0.26 | 0.161 |  | 0.10 | 0.609 |  | 0.08 | 0.689 |
| SVC(superficial vascular complex) | | | | | | | | | | | |
| VD(vessel density,%) | -0.25 | 0.177 |  | -0.13 | 0.495 |  | 0.13 | 0.553 |  | 0.15 | 0.429 |
| PA(perfusion area,mm²） | -0.30 | 0.112 |  | -0.21 | 0.269 |  | 0.10 | 0.607 |  | 0.16 | 0.397 |
| PC(perfusion capacity） | -0.20 | 0.294 |  | -0.42 | **0.021** |  | -0.14 | 0.449 |  | 0.04 | 0.828 |

Abbreviations: iERM, idiopathic epiretinal membrane; BCVA, best-corrected visual acuity; logMAR, logarithm of the minimal angle of resolution; RT, retinal thickness.
